# Improving nutritional status after allogeneic stem cell transplantation: results of phase 2 ALLONUT clinical trial

**DOI:** 10.1038/s41409-024-02271-w

**Published:** 2024-03-20

**Authors:** Sophie Estran, Michael Loschi, Sami Benachour, Alizée Soldati, Edmond Chiche, Rinzine Sammut, Guillaume Robert, Arnaud Jacquel, Jacques Chibois, Stephane Schneider, Thomas Cluzeau

**Affiliations:** 1 Hematology department, Nice University hospital, Cote d’Azur University, Nice, France; 2 INSERM U1065, Mediterranean center of molecular medicine, Nice, France; 3 Bastide Saint Antoine, Grasse, France; 4 Nutrition department, Nice University hospital, Cote d’Azur University, Nice, France

**Keywords:** Quality of life, Health services

## Abstract

Malnutrition increases the risk of non-relapse mortality after allogeneic stem cell transplantation (aHSCT). Here are the results of the ALLONUT clinical trial designed to improve the nutritional outcome of patients receiving aHSCT. ALLONUT is a prospective open label phase 2 clinical trial assessing the efficacy of a close tailored nutritional support and management with traditional and original solutions to improve patients nutritional status following aHSCT. Nutritional status evaluation was performed before transplantation, on Day 0, 30, 100 and one year after transplantation. The study involved 70 patients treated by aHSCT. 10% of patients were moderately or severely malnutrition at baseline and 26.9 were severely malnutrition at D30. Patients’ nutritional status improved thanks to the cooking classes and the personalized outpatient nutrition program. At D100, 23% were still malnutrition, while only 10.8% were severely malnutrition one year after transplantation. The QLQ-C30 show that quality of life (QoL) decreased until D30, and improve to reach the pre-transplant level on D100 before exceeding it on D360. The study confirmed that a close, personalized nutritional program combining traditional and original measures can improve both nutritional status and QoL for patients suffering from moderate or severe malnutrition after aHCST.

## Introduction

Allogeneic stem cell transplantation (aHSCT) is the best curative option for many hematological malignancies. However, its power is hampered by its toxicity, encompassing an uncontrolled inflammatory state, graft versus host disease (GVHD) and infections [1–5]. In recent years, the development of less toxic conditioning regimen, the improvement of pre and post transplantation anti-tumor therapies and supportive cares have made aHSCT available to more patients especially those with an older age and comorbidities [6, 7]. The constant rise of haploidentical transplantation has made the process available for virtually all patients requiring a transplantation with a survival rate comparable to the one of matched unrelated donors [8]. Malnutrition is a frequent complication of the treatment of hematological malignancies [9–13]. The causes are multifactorial including disease associated inflammation, malabsorption, high dose chemotherapy responsible for mucosal damages, nausea, vomiting, diarrhea, dysbiosis, mucositis, dysgeusia, fever, infections. All these side effects lead to a significant reduction in oral intake leading to weight loss and sarcopenia. For patient receiving transplantation the malnutrition status is aggravated by the effects of the conditioning that includes total body irradiation, high-dose chemotherapy and post transplantation complications such as graft versus host disease, steroids and immune-suppressors use. A decreased oral intake also leads to a lower quality of life and poorer survival [14, 15]. The results of a prospective study are presented here, assessing the efficacy of a close tailored nutritional support and management with traditional (enteral and parenteral nutrition) and original solutions (cooking classes) to improve patients nutritional status following aHSCT. The objectives were to decrease the prevalence of malnutrition at day 100 and improve appetite, quality of life and overall survival after ASCT.

## Methods

### Study design

**Fig. 1:**
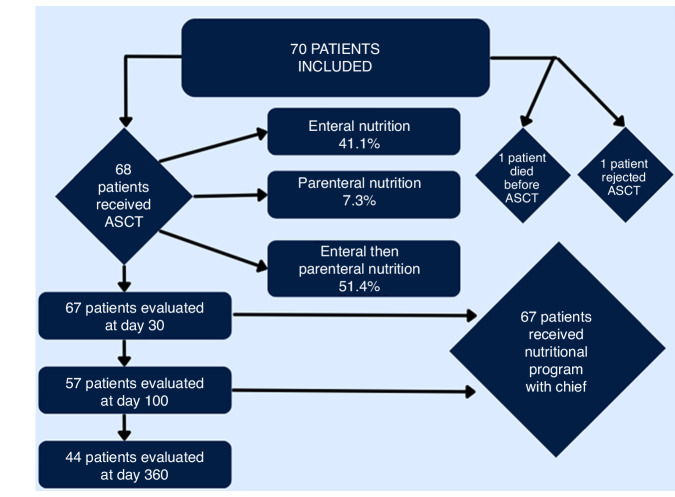
Flow chart of the study.

The ALLONUT trial (ClinicalTrials.gov Identifier: NCT03829072) was a prospective phase II trial conducted at the hematology Nice center to assess the efficacy of a combined approach to treat malnutrition after transplantation. The study enrolled adult patients over 18 years old who underwent an aHSCT between March 2019 and January 2022. All patients received enteral or parenteral nutrition during their hospital stay guided by a weekly dietician evaluation, followed by three cooking classes delivered by a Michelin starred chef (Fig. 1). The study was approved by the national ethics committee called Comité de protection des personnes (CPP AU-1469) referenced ID-RCB 2018-A01842-53, and respected the declaration of Helsinki. All patients signed a written informed consent.

### Malnutrition

The diagnosis of malnutrition according to the French Health Administration [16] was based on the on the European Society for Clinical Nutrition and Metabolism (ESPEN) and GLIM guidelines [17] and required at least one of the following criteria: BMI < 18.5, a weight loss of more than 5% over the last month or more than 10% over the past 6 months. A moderate malnutrition (MM) was defined as a BMI between 17 and 18.5, a weight loss of more than 5% but less than 10% over the last month or more than 10% but less than 15% over the past 6 months and an albumin level lower than 35 g/L but higher than 30 g/L. The diagnosis of severe malnutrition (SM) required all three following criteria a BMI < 17, a weight loss of more than 10% over the last month or of more than 15% over the past 6 months and an albumin level lower than 30 g/dL.

### Nutritional management strategy

**Fig. 2:**
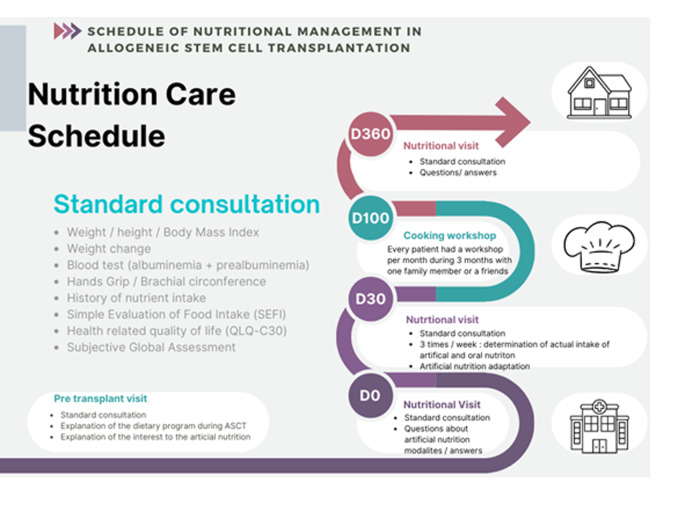
Schedule of nutritional management.

French society of cellular therapy (SFGMTC) recommended nutritional assessment one time before aHSCT and one per week during hospitalization (Fig. 2). After discharge, patients need to be assessed one and three months after aHSCT [18]. It relied on a systematic enteral nutrition starting on day 0. Weekly nutritional assessment allowed for a better adapted nutritional support between the first day of conditioning and day 100 after aHSCT. Oral intakes were calculated and compared to daily calories recommendations for same sex and age in the healthy population. For malnourished patients and patients unable to eat enough to cover their caloric needs, a nutritional intervention was initiated. For most patients the intervention consisted in increasing the daily volume of enteral solution. Several times a week the dietician adapted the nutritional support to the nutritional assessment and the presence of gut acute graft versus host disease (aGVHD). After being discharged from the transplantation unit all patients enrolled in the study benefited from 3 personalized classes with a one-star chef. The chef designed recipes respecting special food guidelines for transplanted patients.

### Main and secondary objectives

The main objective was to show an improvement of nutritional status based on French Health Administration guidelines [16] using ALLONUT clinical trial. The secondary objectives was to show an improvement of nutritional status based on Simple Evaluation of Food Intake (SEFI) and Subjective Global Assessment (SGA) scores, brachial circumference determination, oral intake calculation (calories), albumin and prealbumin blood levels; and an improvement of quality of life based on EORTC QLQC30.

### Endpoints

The evaluation of pre and post transplantation nutritional status relies on a combinatorial of several parameters including Body Mass Index (BMI), weight loss, sarcopenia, hands grip test, albumin level. French guidelines [16] for the assessment of nutritional status include monitoring of these parameters and calculation of several scores such as the SEFI and SGA scores, brachial circumference determination, oral intake calculation (calories), albumin and prealbumin blood levels.

Nutritional management consisted in a first pre-transplant visit with a dietician specialized in the follow up of hematology patients. The goal of this visit was to perform a first nutritional screening based on the ESPEN guidelines [19, 20] and the French Health Administration Guidelines [16] and French Society for Cell Transplantation and Cell Therapy [18]. During the visits, the dietician collected the following values, weight, height, BMI, brachial circumference and Hands grip test, calculated the weight change between the baseline weight at diagnosis and before transplantation, the SEFI and SGA scores, the dietician also collected the most recent history of nutrients intake, and measured albumin and prealbumin blood levels and made the QLQ C30. During this first visit the patient also received explanations on the trial, especially on the nutritional management that would results from nutritional assessment after each visit.

The primary endpoint was the prevalence of malnutrition (SM + MM) at day 100, while secondary endpoints were oral intake (calories), EVA appetite, SEFI score, SGA score, quality of life, brachial circumference and overall survival at day 30, day 100 and day 360 after aHSCT.

### Sample size

We included 70 patients based on the number of Allo-transplanted patients per year in the Nice University Hospital.

### Statistical analysis

Continuous variables are described using medians [interquartile ranges] (minimum; maximum) and qualitative variables are described using counts and percentages. Noncontinuous variables were compared by the chi-square test. Mann–Whitney test was used for continuous variables. The median follow-up time was calculated as the median time from initiation of treatment to last follow-up. Overall survival (OS) was calculated from the date of AML diagnosis to the date of death or last follow-up. Survival curves were estimated using the Kaplan–Meier method and were compared with the log-rank test. Statistical tests were considered significant when the 2 tailed *P* value was <0.05. Confidence intervals (CI) were computed with 95% coverage. All statistical analysis was performed using SPSS v.26 software (IBM SPSS Statistics).

## Results

Between March 2019 and January 2022, 70 consecutive patients who received an aHSCT in hematology Nice center were enrolled in the ALLONUT phase II clinical trial. Patient’s characteristics at baseline are described in Table 1. Sex ratio was well balanced, median age was 55 years old (range, 18–73). The main indication for transplantation was acute myeloid leukemia (56.5%). Most patients received a reduced toxicity conditioning. At transplantation mean BMI was 23.9 with normal prealbumin and albumin levels. Median SGA and SEFI scores were also in the normal range at baseline. Most patients (92.5%) developed aGVHD including 39% with grade III-IV aGVHD after aHSCT.

**Table 1:** Population characteristics.

	*N* = 70 (%)
Sex M/F	34/36
Median Age (range	55 (18–73)
Disease	
- AML	39 (56.5%)
- CML	3 (4.3%)
- Lymphoma	9 (13%)
- Bone marrow failure	2 (2.8%)
- MDS/MPN	16 (23.1%)
CONDITIONING	
TBI	13 (18,8%)
Myeloablative	18 (26%)
HLA matching	
- Identical sibling	12 (17.3%)
- Unrelated 10/10	44 (63.7%)
- Mismatched unrelated	13 (18.8%)
Acute GVHD	
- Grade I	16 (23.1%)
- Grade II	21 (30.4%)
- Grade III	17 (24.6%)
- Grade IV	10 (14.4%)
Chronic GVHD	33 (47.8%)
CMV status	
Positive	37 (53.6%)
negative	32 (46.3%)
Median BMI (range) kg/m²	23.9
	(16–44.9)
Median SGA (range)	2
	(0–22)
Median Albuminemia (range) g/L	43
	(28–54)
Median Préalbuminemia (range) Mmol/L	0.282
	(0.165–0.499)
Median Handsgrip (range) kg	30
	(14–54)
Median brachial circumference (range) cm	30
	(21–40)
Median EVA appetite (range)	10
	(2–10)

### Evolution of nutritional status

At day 0, the prevalence of MM was significantly higher than before aHSCT with 10.3% of MM (*p* = 0.004) (68 patients evaluable). However, after dietary intervention and cooking classes, the incidence of MM was not different than before transplantation at day 30 (67 patients evaluable), day 100 (57 patients evaluable) and day 360 (44 patients evaluable) after aHSCT. The same results were obtained for SM with a significantly higher incidence of SM at day 0 (*p* < 0.0001) and no significant difference at day 30, day 100 and day 360 (Table 2).

**Table 2:** Evolution of moderate and severe malnutrition during aHSCT.

	% of moderate malnutrition (MM)	*p*-value (X²)
Pre ASCT (70/70 patients)	10	
D0 (68/70 patients)	10.3	0.004
D30 (67/70 patients)	17.9	0.472
D100 (57/70 patients)	21.4	0.653
D360 (44/70 patients)	11.6	0.651
	**% of severe malnutrition (SM**	***p*-value (X²)**
Pre ASCT (70/70 patients)	10	
D0 (68/70 patients)	13.2	<0.0001
D30 (67/70 patients)	26.9	*0.132*
D100 (57/70 patients)	23.2	*0.188*
D360 (44/70 patients)	11.6	*0.456*

### Evolution of calories uptake

**Fig. 3:**
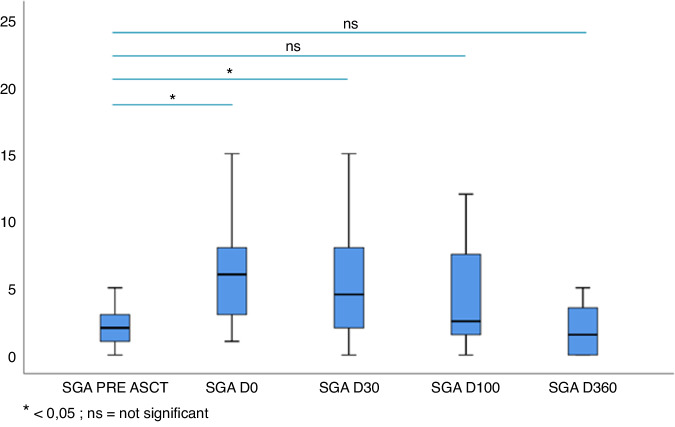
Evolution of SGA score after aHSCT.

**Fig. 4:**
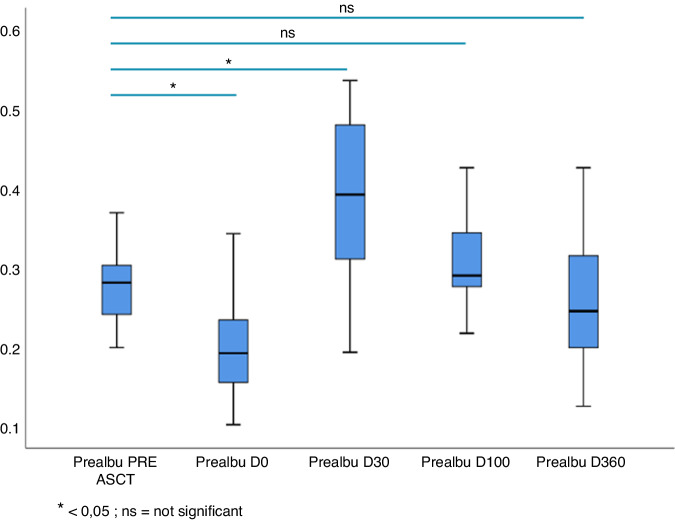
Evolution of pre-albumin after aHSCT.

The food intake measured by calories intake calculation demonstrated an improvement over time with a significantly lower calories intake at day 0 (1653 kcal vs 1045 kcal, *p* = 0.002) and at day 30 (1653 kcal vs 1498 kcal, *p* = 0.02). The oral intake recovered during the trial to reach the pre-aHSCT from day 100. The SGA score followed the same pattern with a significantly higher median SGA score at day 0 (7) than at baseline (2) (*p* = 0.009). The median SGA score gradually decreased at day 30 (*p* = 0.025), day 100 (*p* = not significant) and 360 (*p* = not significant) following aHSCT and was not different from the pre-aHSCT median SGA from day 100 (Fig. 3). The oral intake correlated with the improvement of albumin and prealbumin levels after aHSCT. Albumin and prealbumin levels were significantly lower at day 0 than at baseline with a mean albumin level of 42.2 g/L before aHSCT that decreased to 32.5 g/L at day 0 (*p* < 0.0001). There was no difference in albumin concentration between baseline and day 30, day 100 and day 360 following aHSCT. Regarding mean serum prealbumin level, it was significantly lower at day 0 (0.2 g/L) than before aHSCT (0.3 g/L) (*p* < 0.0001) (Fig. 4). Similar to the serum albumin level, the prealbumin increased at day 30 (0.4 g/L), day 100 (0.3 g/L) and day 360 (0.3 g/L) (data not shown).

### Evaluation of appetite

**Fig. 5:**
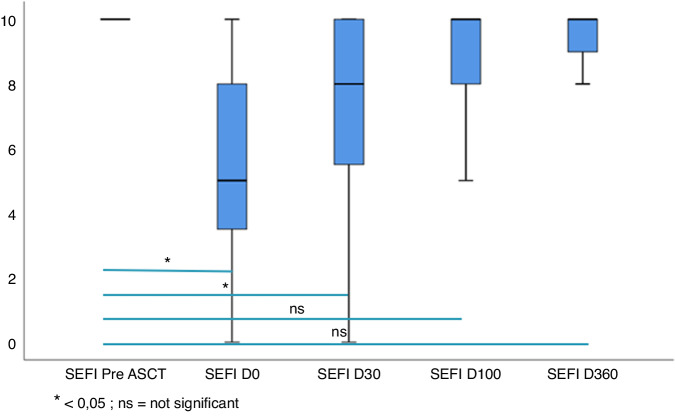
Evolution of SEFI score after aHSCT.

The evaluation of appetite using the SEFI score revealed an improvement of the score over time after aHSCT. At day 0, patients had a significantly lower appetite as compared to pre-transplantation baseline (5 vs 10, *p* < 0.0001). SEFI score stay significantly different from baseline at day 30 (*p* < 0.0001) and appetite was restored from day 100 with no significant difference observed at day 100 and day 360 (Fig. 5).

### Evaluation of brachial circumference and hand grip test

Muscle mass and function evaluation through brachial circumference (BC) and hand grip test revealed no difference for the mean BC at day 0 with a mean BC of 30 cm at baseline versus 39 cm at day 0, 28.1 cm at day 30, 28.4 cm at day 100 and 28.9 cm at day 100. There was also no difference for mean hand grip test result between pre and post aHSCT results (data not shown).

### Evaluation of quality of life and survival

**Fig. 6:**
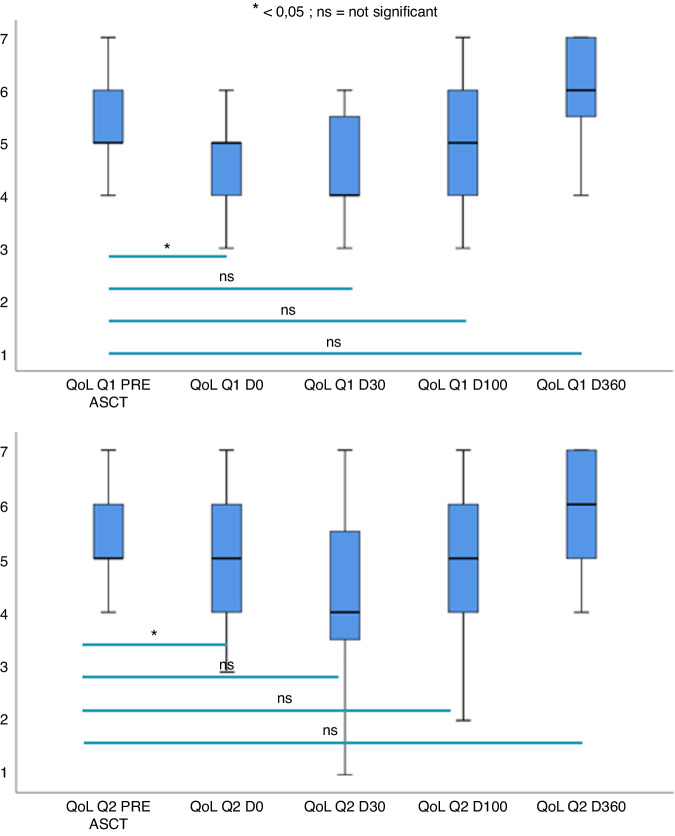
Evolution of quality of life after aHSCT.

**Fig. 7:**
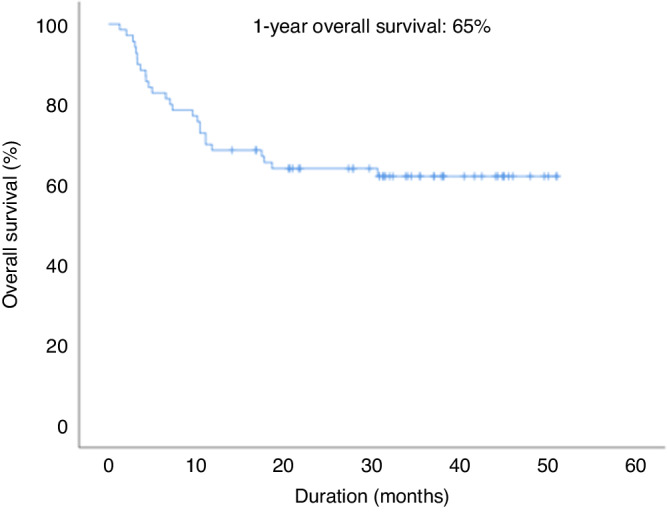
Overall survival in ALLONUT clinical trial.

The quality of life was significantly worse only at day 0 than before aHSCT (*p* = 0.002). Figure 6 showed evolution of quality of life based on question 1. *(How would you rate your overall health during the past week?*) and question 2 (*How would you rate your overall quality of life during the past week?*) of QLQC30. Quality of life was restored from day 30 in the study. One-year overall survival was 65% (Fig. 7).

## Discussion

Malnutrition has been the focus of recent investigations as it has been shown to correlate with weight loss, non-relapse mortality and therefore overall survival after aHSCT. Several factors contributed to malnutrition before, during and after transplantation. In the pre aHSCT period, chemotherapy, inflammation caused by the underlying disease and infections lead to a decreased balance of calories intake and a catabolic state [21–23]. In the study, 20% of the patients already suffered from malnutrition at baseline. Conditioning regimen and severe aGVHD (especially gut aGVHD) are risk factors for the onset of severe malnutrition. In the clinical trial, most patients displayed symptoms of aGVHD, among whom 39% had severe grade III-IV aGVHD.

Despite the current effort in standardizing the evaluation of malnutrition for aHSCT, a unique scoring system has yet to be validated. Several systems have been proposed to better assess the severity of malnutrition. An early and dynamic evaluation of nutritional status in cancer patients is provided by the Patient Generated Subjective Global Assessment (PG-SGA), that is recommended by the Academy of Nutrition and Dietetics [24]. The higher the score, the worse the nutritional status is. Serum albumin and prealbumin levels are commonly used as biomarkers to both diagnose and follow patients’ nutritional status after aHSCT and holds a predictive value for those suffering from gut aGVHD. In the study, SGA and biomarkers including serum albumin and pre-albumin levels moved in parallel after aHSCT showing an initial alteration followed by a recovery from day 30. The impact of malnutrition on quality of life was assessed using the QLQC30 and showed a decrease in quality of life in parallel with the level of malnutrition. A recent prospective study on 36 aHSCT recipients reported a link between malnutrition and quality of life [25]. In this prospective cohort a severe malnutrition at the time of discharge after aHSCT was correlated with a worse quality of life. At day 360, transplanted patients had better quality of life than before aHSCT partly due to complete remission status and free of treatment for hematologic malignancy.

Current management of malnutrition varies between countries and even between transplant centers in a same country. To homogenize nutritional management of cancer patients, the French Health agency published its guidelines [18]. These recommendations are based on the ESPEN guidelines and stratify malnourished patients into MM or SM. A recently reported European multicenter retrospective study has reported the current practices in nutrition in 28 transplant centers from Austria, Germany and Switzerland [26]. The authors reported that all centers had national nutritional guidelines for aHSCT recipients. Seventy-five percent of centers used parenteral nutrition (PN) based on a drop of oral intake or body weight and adapted the dose of PN to the remaining oral intake. The Francophone Society for Bone Marrow Transplantation and Cellular Therapy also provides nutritional guidelines for transplantation centers [18]. Based on that PN could increase catheter infections and favorize gut dysbiosis increasing risk of gut aGVHD, frontline enteral nutrition (EN) was used for all patients at day 1 after aHSCT with a dose adapted three times a week to the remaining oral intake based on dietitian evaluation.

Malnutrition most often persists and can aggravates after aHSCT related to loss of appetite, dysgeusia, aGVHD and many restrictions for cooking. All patients received a personalized cooking classes with a one-star chef. He designed 4 to 5 courses meal respecting patients’ restrictions after aHSCT. The SEFI score was assessed at baseline, day 0, day 30, day 100 and day 360 after aHSCT and showed a restoration of appetite from day 100 after aHSCT when they have completed all the nutritional program.

The study reports an original approach to improve the nutritional state after aHSCT combining both the screening of malnutrition, a tailored nutritional support adapted several times a week based on clinical and biological tests, but also using cooking classes to improve appetite and food intake. In this study, there was a decrease of malnutrition at day 30 compared to the literature. This decrease of prevalence of malnutrition at day 30 was associated with a faster than usual improvement for recovery of appetite and quality of life. Even if it was not clearly observed in the study due to the small number population, improvement in nutritional status suggests that it could have an impact on immune recovery and GVHD rates after HSCT. The absence of a control group and the size of the sample limits the power of this study and the approach requires a validation in larger multicentric prospective clinical trial. The ALLONUT program could be implemented in all transplant units to be evaluated in a larger real-life cohort. Cooking classes will be edited as a cooking book to become available for all transplanted patients in France.

## Conclusion

The ALLONUT clinical trial combined an original, never reported dual approach to tackle malnutrition after aHSCT, using three times a week dietitian adapted nutritional support to improve calories intake and cooking classes with a one-star chef. We have assumed that the use of this personalized nutrition program could induce an early restoration of malnutrition, appetite and quality of life.

### Supplementary information

Supplemental Table 1

## Data Availability

Dataset is available on demand to corresponding author.
